# Supplementary material for: Rise of the war machines: Charting the evolution of military technologies from the Neolithic to the Industrial Revolution
Source: PLoS One. 2021 Oct 20;16(10):e0258161. doi: 10.1371/journal.pone.0258161 (PMC8528290; doi:10.1371/journal.pone.0258161)
Supplement: S1 File — (DOCX) [file pone.0258161.s001.docx]

Supporting Information for Rise of the War Machines: Charting the Evolution of Military Technologies from the Neolithic to the Industrial Revolution

**Authors**

Peter Turchin^2,3,11^, Daniel Hoyer*^† 1,4^, Andrey Korotayev^5,6^, Nikolay Kradin^7^, Sergey Nefedov^8^, Gary Feinman^9^, Jill Levine^1^, Jenny Reddish^2^, Enrico Cioni^11^, Chelsea Thorpe^11^, James S. Bennett^10^, Pieter Francois^11^, Harvey Whitehouse^11^

**Affiliations**

^1^ Evolution Institute. Tampa, USA.

^2^ Complexity Science Hub. Vienna, Austria.

^3^ University of Connecticut. Storrs, USA.

^4^ George Brown College. Toronto, Canada.

^5^ HSE University, Moscow, Russia.

^6^ Institute of Oriental Studies. Russian Academy of Sciences, Moscow, Russia.

^7^ Institute of History, Archaeology and Ethnology, Far East Branch of the Russian Academy of Sciences. Vladivostok, Russia.

^8^ Institute of History and Archeology of the Ural Branch of the Russian Academy of Sciences, Ural Federal University. Yekaterinburg, Russia.

^9^ Field Museum of Natural History. Chicago, USA.

^10^ University of Washington. Seattle, USA.

^11^ University of Oxford. Oxford, England.

^*^  Corresponding author. Contact: [dhoyer@evolution-institute.org](mailto:dhoyer@evolution-institute.org)

# Supplementary Methods

## A Brief Introduction to the Seshat Global History Databank

Our method of quantifying global history utilizes [*Seshat: Global History Databank*](http://seshatdatabank.info/), a novel system for gathering and storing information on the human past relating to a wide range of topics, including social organization, religion, ritual, warfare, and economic life over roughly 10,000 years of world history. The data underpinning this study were gathered in line with Seshat’s robust data architecture and a transparent workflow. The basic unit for which data are gathered is the polity, which we define as any independent political unit ranging from archaeological cultures villages (local communities) through simple and complex chiefdoms to states and empires, regardless of degree of centralization (see the project [page](http://seshatdatabank.info/methods/code-book/); further detailed in 1, 2). Currently, the Seshat project does not have data for all historical polities. Instead, during early phases of the project, we assembled a stratified sample of polities using the concept of “NGA” (Natural Geographic Area). An NGA is a fixed spatial location of roughly 100 x 100 km delimited by naturally occurring geographical features (for example, a river basin, a coastal plain, a mountain valley, or an island). All polities that occupied the NGA, or part thereof, at a century mark (e.g. 200 CE, 300 CE, 400 CE), are included in our sample. This strategy avoids oversampling (redundantly repeating information across time points) while still capturing meaningful changes in the variables of interest.

Where NGAs contain many small-scale polities (e.g. local chiefdoms) during particular periods it is not always feasible to code information separately for each polity. In such cases we treat local groups sharing a common ethnological “culture” (3, 4) or an archaeological sub-tradition (5) as a single ‘quasi-polity’, on the assumption that most of the information applying to any one constituent group applies equally to all of them. This method is particularly helpful for coding data on prehistoric polities whose boundaries are unknown. Nevertheless, whenever evidence of variation within polities or quasi-polities is available, we attempt to capture that, as well as evidence of changing cultural boundaries, spheres of political influence, or provenance of political control.

To maximize diversity within our sample of polities we initially divided the globe into 10 world regions and for each of these regions we selected 3 NGAs: one NGA where social complexity arose early within that world region, one NGA where it arrived late, and one NGA where it arrived at an intermediate point in time (see Fig 1 of main text). Our intent is to maximize diversity in the sample rather than skewing the data towards the more extensively studied regions of the world where social complexity emerged early.

Since then, we have continued to add further polities from different regions to the initial sample; the data included in this study comes from 373 historic polities covering 35 NGAs. For a full list of both NGAs and polities, see the latest data release (6) and the Seshat [Data Browser](http://seshatdatabank.info/databrowser/). Crucially, this strategy to sample polities using the NGA concept allows us to organize our data in time-series and analyze the data for dynamical patterns.

## Coding Historical Information into the Seshat Databank

Within each polity, data is gathered for a growing number of variables. The [Seshat Codebook](http://seshatdatabank.info/methods/code-book/) specifies the full list of variables, including the specific variables used in our analyses for this paper. Variables are selected and data are gathered as part of a process in which experts (recognized authorities on the polity in question, typically holding a relevant doctorate and occupying a faculty position in a university) and fully trained research assistants (RAs) play vital and complementary roles. First, for each paper an initial list of variables is put together which reflects the different hypotheses under review. Input by experts first focuses on providing help with generating this initial variable list, or Coding Scheme, as well as assembling reading lists and, where necessary, advice on how to interpret some of the key historiographical debates.

Once the variables in the Coding Scheme have stabilized, full scale data collection occurs in a stepwise process. RAs gather the information necessary to put forward a provisional coding recommendation, together with a condensed overview of the data used to buttress that coding, highlighting any areas of uncertainty. These codes are based on scholarly sources and fully referenced. While compiling information, the RAs note where information is lacking or ambiguous and periodically consult with the Seshat experts on these matters. Seshat experts are thus involved in reviewing the data, addressing questions of interpretation, filling gaps or confirming that data are unavailable. The names of both Seshat experts and RAs are linked to the data. This information on expert provenance and keeping a log of the dates of their interventions help us to assess the state of maturity of the data curation process for any given variable.

In addition to the codes of ‘absence’, ‘presence’, and ‘unknown’, a coding of ‘inferred’ absence (or presence) is used when direct evidence for a particular variable was sparse or lacking but indirect evidence made clear that it was more likely to have been absent (or present) than not. This approach avoids a situation in which researchers inaccurately coded the trait ‘unknown’ when in fact what was known was more than nothing. In addition, variables could be coded as first absent but then present during transitional periods or could be coded in multiple ways simultaneously where experts disagreed, thus providing grounds for more than one coding outcome. When Seshat experts pointed out disagreements in the literature or disagreed among themselves on a particular coding, we kept a record of this so that multiple analyses could be run taking into account contrasting interpretations. For more information on the levels of uncertainty and disagreement, see (7).

For each variable we store three types of information. First, we gather machine-readable values. Rather than using an arbitrary scale to code features that vary in magnitude, we prefer to quantify variables (e.g. estimated population size) or fractionate them into multiple features that can be coded as either absent or present (allowing also for ‘inferred’ codes). Second, if applicable, we gather as much information as possible on levels of uncertainty and disagreement as expressed within the literature or amongst experts. As we capture this information in machine readable format, we can include this information in our analyses. Third, Seshat also contains narrative paragraphs explaining the rationale for coding each variable a certain way. This text is typically drafted by a research assistant, often quoting from authoritative sources, and is later checked, edited, and augmented by Seshat experts. Wherever possible, information in Seshat is linked to fully cited sources which, in addition to scholarly publications, may include personal communications from one or more Seshat experts.

Before the data gathered in this way is used for analysis, a process of extensive data quality checks is undertaken whereby all machine-readable data is checked for syntax validity, completeness and trustworthiness of sources. Since our knowledge of the past is constantly evolving and the data coded needs to be augmented incrementally, we also publish our data online after it has been used in publications and invite the scholarly community to scrutinize and edit it so that the vetting process is ongoing. With the release of our Equinox2020 dataset (<http://seshatdatabank.info/databrowser/>) we are now doing so for preprints as well.

## Aggregation of Seshat Measures into Predictor Variables

During the data collection stage complex variables are broken down into simpler components and data are gathered for each component. Before analysis we assemble simpler (often binary) variables into more quantitative measures that are more suitable for statistical analysis. We illustrate this process by showing how we have constructed the Information Complexity variable (Info for short; variables are italicized for readability) which is used in a number of Seshat publications.

*Info* is based on 15 binary Seshat variables. The first four provide the basis for measuring the sophistication of the writing system (following the description of the variables in the Seshat codebook):

- **Mnemonic devices** such as tallies
- **Non-written records**, which are more extensive than mnemonics, e.g., quipu
- **Script** as indicated at least by fragmentary inscriptions (note that if written records are present, then so is script)
- **Written records**: these are more than short and fragmentary inscriptions (such as those found on tombs or runic stones). There must be at least several sentences strung together. For example, royal proclamations from Mesopotamia and Egypt, which can be quite lengthy, qualify as written records

These binary measures are combined to produce a Writing scale from 0 to 4:

- 0 = no evidence of writing for the coded polity
- 1 = only evidence of mnemonic devices is present
- 2 = non-written records are present, but no script
- 3 = evidence for script in fragmentary inscriptions, but no lengthy texts
- 4 = written records are present

Presence or absence of a “less sophisticated” writing variable doesn’t affect this scale (so if “script” is present, it does not matter whether non-written records are present or absent).

The next two Seshat variables code for whether the writing system is phonetic (e.g., alphabetic) or non-phonetic (e.g., ideographic). They are not used in the scale for Info, as they only code for the type of script.

The next nine binary variables code for presence or absence of various kinds of Texts:

- **Lists, tables, and classifications**, as used in trade
- **Calendar**
- **Sacred Texts**, which originate from supernatural agents (deities), or are directly inspired by them
- **Religious literature**, which differs from the sacred texts in that it provides commentary on the sacred texts, or advice on how to live a virtuous life
- **Practical literature**: for example, manuals on agriculture, military, cooking, etc.
- **History**
- **Philosophy**
- **Scientific literature** including mathematics, natural sciences, social sciences
- **Fiction** including poetry (but must be written down)

The variable texts, which scales from 0 to 9, sums the number of securely attested types of texts (that is, coded as “present”). The idea here is that the more sophisticated a society is informationally, the more different types of texts it will have in circulation. For this reason, we only count those types of texts that were definitely in circulation and left clear evidence of their use (in other words, “absent”, “unknown”, or even “inferred present” do not constitute such strong evidence of presence). Finally, to construct Info we simply sum Writing and Texts scores. Thus, Info can vary between 0 and 13.

It is important to note that the above scheme is only one of the possible ways to come up with a quantitative measure of information sophistication. Other analysts are free to combine and recombine Seshat variables in different ways. One of our goals, when designing the conceptual approach used in Seshat, was to separate data coding and data analysis steps as much as possible, providing analysts with freedom to define entities of interest that are most suitable to their analysis goals.

## Defining the Response Variable: coding military technologies in past societies

As described in the main text, the primary response variable used in analyses was a measure of Military Technologies (MilTech), a combination of six ‘Warfare Characteristics’ (WCs) that, in turn, are composed of 46 unique variables defined in the Seshat Codebook. These are:

**WC1: Metals**

1. **Military use of copper**
2. **Military use of bronze**
3. **Military use of iron**
4. **Military use of steel**

**WC2: Projectiles**

1. **Javelin,** including thrown spears
2. **Atlatl**
3. **Sling**
4. **Self bow,** a bow made from a single piece of wood (e.g. the English/Welsh longbow)
5. **Composite bow,** a bow made from several different materials, usually wood, horn, and sinew, also known as a laminate bow
6. **Crossbow**
7. **Tension siege engine,** e.g. catapult or onager
8. **Sling siege engine,** e.g. trebuchet
9. **Artillery,** e.g. canon, mortar shell
10. **Handheld firearms,** e.g. muskets, pistols, and rifles

**WC3: Weapon**

1. **War Club**
2. **Battle Axe**
3. **Dagger**
4. **Sword**
5. **Spear**
6. **Polearm**

**WC4: Armor**

1. **Wood,** including bark
2. **Leather or cloth**
3. **Chainmail**
4. **Scaled,** armor consisting of many individual armor ‘scales’ or plates
5. **Laminar,** armor made from horizontal overlapping rows or bands of solid armor plates
6. **Plate,** armor made from iron or steel plates
7. **Shield**
8. **Helmet**
9. **Breastplate,** any form of torso protection
10. **Limb protection,** protection for arms, legs, or both, e.g. greaves

**WC5: Military Animals**

1. **Donkey** used in military operations
2. **Llama** used in military operations
3. **Elephant** used in military operations
4. **Camel** used in military operations
5. **Horse** used in military operations

**WC6: Defensive Fortifications**

1. **Settlements** in a defensive position, settlement location chosen for defensive reasons, e.g. on a hill top, peninsula
2. **Wooden palisades**
3. **Earth ramparts**
4. **Ditch**
5. **Moat,** which differs from a ditch in that it has water
6. **Stone walls (non-mortared)**
7. **Stone walls (mortared)**
8. **Fortified camps,** camps made by armies on the move, e.g. on a campaign
9. **Complex fortifications,** fortifications featuring two or more concentric walls
10. **Long walls,** fortifications used not to protect a specific city or town, but a large territory, e.g. the Great Wall(s) of China
11. **Modern fortifications,** those made after the introduction of gunpowder, e.g., trace italienne/starfort.

These variables are coded as binary measures (absent or present, allowing for coding inference and unknown as well) following the general Seshat procedure described above. First, following the approach that we have introduced and validated in a previous article (2) we aggregate these 46 variables into the six defined WCs, which are summed to produce our single continuous MilTech measure.

Note that we do not include specifically maritime military technology in our MilTech measure. One of the primary goals of this study was to investigate theories about the influence of interstate military competition, and specifically horse-mounted warfare. Terrestrial technologies are most apt to this investigation and so are the focus of the present work. Further, the warfare characteristics we explore here, which includes mainly terrestrial technologies, are particularly suited to broad comparative analyses, since they are shared by a large number of polities in our sample. This is not the case with many maritime technologies. It is notable, though, that several of the technologies in our list were also involved in maritime warfare as well (e.g. the use of iron and steel in ship building, or canon artillery mounted onto ships). An interesting open question for future studies is whether certain marine technologies exhibit similar patterns to the terrestrial ones explored here, and perhaps specifically whether the invention and adoption of ocean-borne vessels created an ‘arms race’ in the early modern period similar to that seen with the spread of cavalry and related technologies in antiquity.

We used two general principles to construct WCs from the Seshat binary variables. First, for most WCs it is possible to arrange the binary variables, on which the WC is based, into a sequence from less “sophisticated” (advanced, effective) to more sophisticated / advanced. This judgment is based on observing which weapons became obsolete as they were replaced by an advanced version. For example, bows (especially the more powerful varieties, such as compound bows and crossbows) replaced thrown spears (javelins, atlatls) as standard army equipment. Later on, handheld firearms replaced bows. Similarly, and for a different WC, iron replaced bronze as the preferred material for weapons and armor. It is understood that this process of replacement was not instantaneous, and that early versions of the more “advanced” military technology could be inferior in some ways to the late, sophisticated versions of a less advanced technology. For example, early firearms were inferior to late versions of composite bows in their rate of fire and range. Yet, eventually firearms entirely replaced bows as military equipment.

This observation is important because for some Seshat composite measures it is appropriate to sum the binary variables, but for others it is not. For example, when constructing the variable Texts (see the previous section) we summed the variety of different types of texts (calendar, religious literature, history, science, fiction, etc). The idea is that the variety of texts can serve as a proxy for the size of the literate population that consumes them. On the other hand, the variable Writing did not sum the binary variables it is based on, but reflected only the most sophisticated one. As we said above, presence or absence of a “less sophisticated” writing variable doesn’t affect this scale. For many WCs it makes sense to follow the same approach. For example, once firearms became standard military equipment, it is irrelevant whether the society in question uses an inferior technology (slings, bows), and thus it’s presence or absence should not affect the level of the Projectiles WC.

The second guiding principle that we used in constructing WCs is the “strong evidence” rule, which works as follows. If an archaeological study finds multiple examples of spears (indicated by spear points) and axes, but no swords, we code spears and axes as “present”, and swords as “inferred absent.” It is quite possible that some rare individuals in the coded society possessed swords (perhaps imported from elsewhere). But if swords are so rare that they didn’t leave a trace in the archaeological record, then they shouldn’t be included in an overall index of MilTech—our aim is to capture weapons that are commonly used.

As an additional note, sophisticated weapons and armor were often high prestige items, and thus were likely to be deposited in, for example, the elite graves. In other words, “absence of evidence” for such implements of war is, indeed, typically strong evidence that they were not widespread. The “strong evidence” rule, which codes such situations as “inferred absent” should be used judiciously, and is not appropriate for many variables other than MilTech. In the case of MilTech, however, it is justified because, as we said above, our emphasis is not on whether the societies in focus knew about a technology, but whether they used it on a relatively massive scale. Furthermore, this rule works well together with the first rule (ranking technologies from less to more advanced). Once a more sophisticated technology is present, the research assistants were instructed not to devote effort to researching whether less advanced version were also present, because their presence or absence doesn’t affect the WC aggregating this aspect of MilTech. For this reason, there are many uncoded/unknown codes that are ignored for the analysis.

A final note that the general procedure described above is only one possible strategy for aggregating binary codes into variables used in the analysis. Below we check how our general results are affected if we don’t use the “strong evidence” rule (*Investigating the Effect of Alternative Coding*). Furthermore, by publishing the raw coded data in the Equinox2020 dataset we encourage scholars to perform analyses using alternate approaches. The specific details of aggregating the 46 binary MilTech variables into the warfare characteristics were as follows:

- **Metal**: max of MilMetal, range = 0–4 (Copper, Bronze, Iron, Steel)
- **Project**: max of MilProj, range = 0–10 (Javelin, Atlatl, Sling, SelfBow, CompBow, CrossBow, TensSiege, SlingSiege, Artillery, HandGun)
- **Weapon**: sum of Weapon, range = 0–6 (WarClub, BattleAxe, Dagger, Sword, Spear, Polearm)
- **Armor**: max of 1. Wood, 2. Leather, 3. Chain/Scale, 4. Lamin/Plate + sum(Shield, Helmet, Breast, Limb), range = 0–8
- **Animal**: max of MilAnimal, range = 0–3 (Donkey or Other=Llama, Elephant or Camel, Horse)
- **Defense**: max of 1. DefPosition, 2. Rampart, 3. NonMStone, 4. Palisade, 5. Stonewall + max of 1. Ditch, 2. Moat + sum(FortCamp, ComplxFort, ModernFort) + (LongWall>0), range = 0–11

As we said above, we construct MilTech by summing these six WCs. Thus, the total range over which MilTech can vary is 0–46. Note that this scheme treats each technology as equivalent, as the adoption of each one adds equally to the final ‘score’ within this range. We check this assumption below by comparing the summed measure of MilTech with an alternative one, based on the first principal component (see *Basic Statistics and Cross-correlations* below). We also perform additional supplementary analyses to subsample these variables randomly, also described below. The primary findings reported in the main text are robust to all of these additional analyses.

# Quantifying Centrality

Several theories argue that advances in technology, especially military ones, may spread more readily to areas connected to each other through information exchange networks (INs) than to more spatially or culturally proximate regions (8-10) (see *Theoretical Background* in the main text for details). For the period under consideration in the present study, the most apparent, and perhaps most significant, IN was the so-called Silk Routes—which, together, “represents one of the world’s preeminent long-distance communication networks” (11). While there has been much scholarly disagreement about where precisely these routes extended and even about what exactly constitutes a ‘silk route’, it is widely acknowledged that together various well-travelled land and sea routes facilitated the exchange of material, people, and ideas between Eastern and Western Eurasia (11-14). Long-distance exchange along certain routes connecting East, Central, and West Asia was active from at least the late 4^th^ millennium BCE; the number of routes and intensity of activity continued to expand during the Ancient and Medieval periods, and while land routes faded in activity after the 16^th^ century CE, sea routes gained momentum and continued to be important conduits of long-distance exchange until the early modern period (and, arguably, to the present day; a useful summary of the evidence for these dynamics is provided by (12)). These Silk Routes, therefore, provide a useful proxy for the central IN theorized to exert an influence over the evolution of MilTech during its Eurasian phase and as it became truly global after 1500 CE.

To construct our measure, we compiled a list of important nodes along various Silk Routes identified by recent scholarship. Specifically, we adopted the list of significant places along various land routes from the [*Digital Silk Road Project*](http://dsr.nii.ac.jp/geography/index.html.en)*,* supplemented by places including those along sea routes listed in the [*UNESCO Cities Along the Silk Roads*](https://en.unesco.org/silkroad/silk-road-themes/cities-silk-roads) project; see Fig 1 in the main text (a complete list of these nodes and their coordinates are provided as Supplemental Files associated with this article). We then established latitude and longitude coordinates for each of these places using Google Earth v.7.3.3 software and calculated the distance from each of our NGAs (measuring from the center-point) to the nearest node along any of the land or sea Silk Routes. In analyses, we use the inverse of this distance measure to determine which NGAs were closest to (or lay within) corridors along these Silk Routes, taking this closeness as a proxy for the Centrality of each NGA to the expanding (and eventually global) IN. Because it is possible that the distance effect is nonlinear, we used the Box-Cox transformation for it. Thus, our measure of Centrality is –*d^θ^* , where *d* is the distance to the nearest Silk Route node and *θ* is the Box-Cox parameter, which was estimated as 0.25.

We also explored other methods for proxying this same connectivity, which are described in detail below; this Centrality measure, however, displayed the most significant effect on the evolution of MilTech, as presented in the main text. Note that while the Silk Route nodes employed here to construct our Centrality measure are taken to proxy the initial Eurasian information exchange network, we assume that this IN was continually expanding over time in roughly concentric waves, until it eventually came to cover the entire globe shortly after 1500 CE. Thus, the Centrality measure signals how regions situated along the long band of central Eurasia extending East-West from roughly Beijing to Venice came to develop similarly high levels of MilTech at an earlier date compared to other regions. These other regions, in turn, became increasingly similar to these ‘early adopter’ regions over time roughly in inverse order to how closely they are situated to this central Eurasian band. This effect is indeed borne out by the dynamic regression analyses and supported by supplementary analyses, detailed below.

# Similarity Analysis

As explained above (see *Quantifying Centrality*), we expect that as NGAs join the expanding (and eventually global) IN, regions that join this IN become increasingly similar to early adopter regions (and to each other). However, our main measure of the sophistication of military technologies, MilTech, is simply an aggregation of 46 binary variables coding for presence or absence of a particular military technology. Thus, the same value of MilTech in two different locations may result from a different mix of technologies. In this analysis we look at a more direct measure of similarity between different geographic regions (NGAs). We calculated similarity between a pair of NGAs as a sum of how many of the 46 variables present in both locations at each time step were the same. As a result, for each time step we calculate (symmetric) similarity matrices, the elements of which tell us how pairwise similarities evolve over time (Fig S1).

For early periods, e.g. 4000 BCE, very few traits are shared between any NGAs. Between 2500 BCE and 1500 BCE, however, we see the development of a second peak in the frequency distribution of similarity, located at values of similarity > 10. This peak becomes larger in successive time periods as more NGAs join the global IN. Finally, by 1500 CE the first peak, located at values of similarity near 0, disappears, with most NGAs having joined the IN. Since the break point between the two peaks appears to be located around similarity = 10, we can trace how NGAs join the IN by noting when they cross 10 to become part of the second “bulge” (see Fig 6 in the main text).


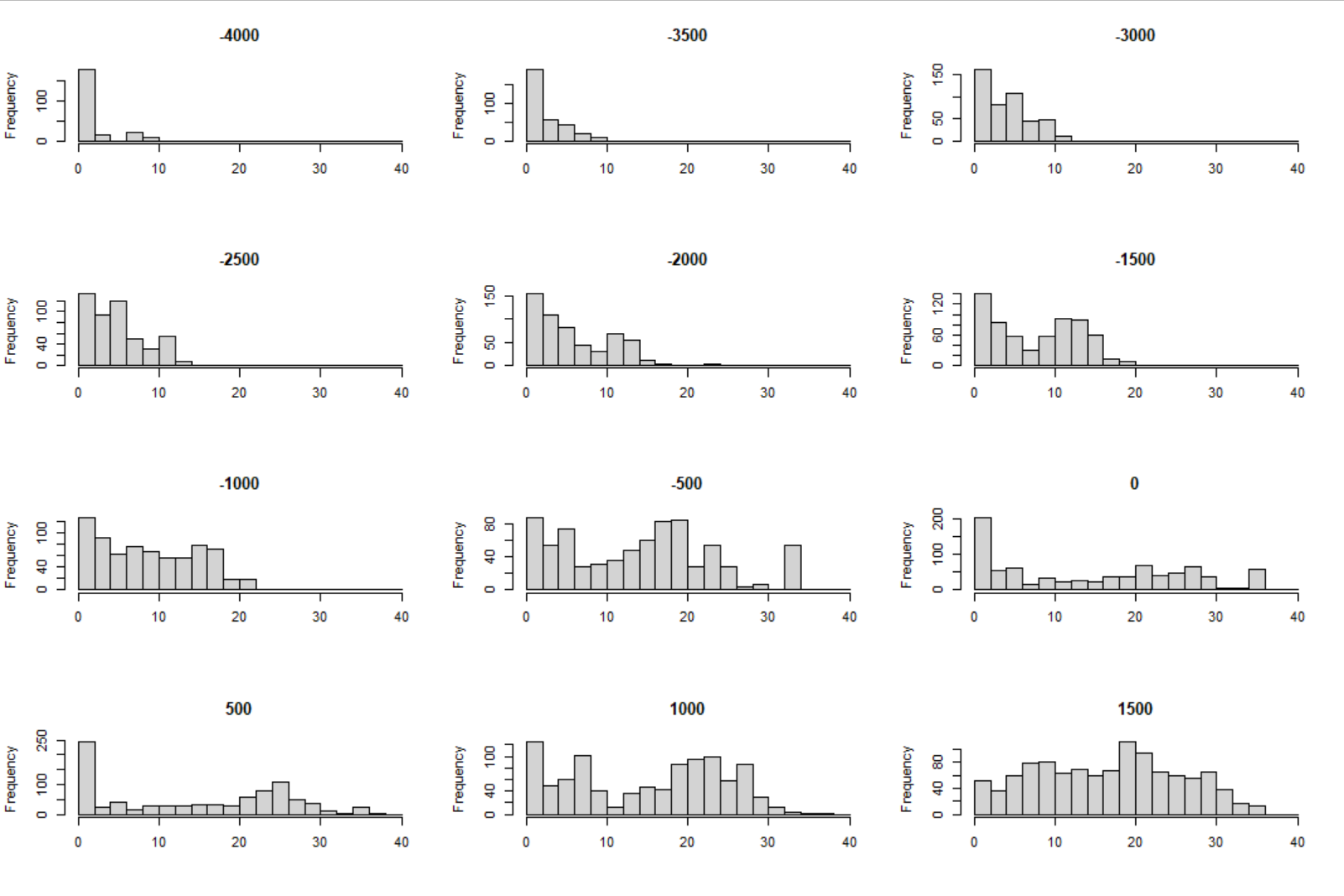


**Fig S1. Frequency distributions of the magnitudes of elements in similarity matrices (showing how many MilTech variables were shared between all pairs of NGAs), shown at 500-year intervals.**

# Assessing Uncertainty Associated with Regression Results

Missing values, estimated uncertainty, and expert disagreement in the predictors (independent variables) were dealt with by multiple imputation (15, 16). The response (dependent) variable, however, is not imputed, because such a procedure can result in biased estimates.

Imputation involves replacing missing entries with plausible values, and this allows us to retain all cases for the analysis. We use the approach of multiple imputation, in which analysis is done on many data sets, each created with different imputed values that are sampled in probabilistic manner. This approach results in valid statistical inferences that properly reflect the uncertainty due to missing values (17). Our procedure followed the approach introduced in (2):

- *Expert disagreement*. In cases where experts disagree each alternative coding has the same probability of being selected. Thus, if there are two conflicting codings presented by different experts and we create 20 imputed sets, each alternative will be used roughly 10 times.
- *Uncertainty*. Values that are coded with a confidence interval are sampled from a Gaussian distribution whose mean and variance are estimated assuming that the interval covers 90 percent of the probability. For example, if a value of [1000–2000] was entered for the polity population variable, we would draw values from a normal distribution centered on 1500 with a standard deviation of 304. Thus, in 10 percent of cases the value entered into the imputed set will be outside the data interval coded in Seshat. For categorical or binary variables we sample coded values in proportion to the number of categories that are presented as plausible. For example, if our degree of knowledge doesn’t allow us to tell whether a certain feature was present or absent at a particular time then the imputed data sets will contain “present” for roughly half the imputed sets and “absent” for roughly half the sets.
- *Missing data*. For missing data we impute values as follows. Suppose that for some polity we have a missing value for variable A and coded values for variables B through H. We select a subset of cases from the full dataset in which all values of A through H variables have values and build a regression model for A. Not all predictors B–H may be relevant to predicting A, and thus the first step is selecting which of the predictors should enter the model. Once the optimal model is identified, we estimate its parameters. Then we go back to the polity (where variable A is missing) and use the known values of predictor variables for this polity to calculate the expected value of A, using estimated regression coefficients. However, we do not simply substitute the missing value with the expected one (because, as explained above, this is known to result in biased estimates). Instead, we sample the regression residuals and add it to the expected value. We applied the same approach to each missing value in the data set, yielding an imputed data set without gaps.

The overall imputation procedure was repeated 20 times, yielding 20 imputed sets that were used in regression analysis as tests for the hypotheses.

Another source of potential bias is the violation of the assumptions of the statistical model needed to calculate confidence intervals and associated *P*-values. Regression diagnostics indicate that the distribution of residuals violates the normality assumption (see *Supplementary Results* below). Furthermore, for any data coming from the same geographic locality (NGA), it is possible that values are not truly independent due to memory effects. While we estimate and, when appropriate, model short-term memory effects by fitting autoregressive terms, there is also a possibility, which cannot be discounted, that there is a longer term memory in the system.

We used nonparametric bootstrap to deal with this problem. Instead of sampling (with replacement) each data point (polity-century), however, we sample with replacement the whole block of data associated with each NGA. Thus, the bootstrap procedure we used mimics the process by which we constructed the Seshat sample (see *A Brief Introduction* above).

We combined multiple imputation with bootstrap by, first, creating 20 imputed data sets, as described above. Second, we resampled, with replacement, NGAs in each imputed dataset 50 times, for a total of 20 x 50 = 1,000 bootstrapped data sets. We then calculated the statistics of interest (regression coefficients associated with various predictors) and constructed the frequency distribution of the 1,000 bootstrapped values. The *P*-value is approximated by the proportion of statistic values greater than 0 (if the hypothesis we test is that the effect of the predictor is positive), or less than 0 (otherwise). The 95 percent confidence interval is then approximated by eliminating the smallest 25 and largest 25 values. Calculating P-values and confidence intervals assuming normality is expected to yield more liberal estimates, while resampling whole blocks of data for each NGA is a more conservative approach. Thus, using these two approaches permits us to bracket the true values.

The analysis sequence follows a two-phase approach. In the first phase we check multiple assumptions about which autoregressive terms need to be explicitly modeled, which predictors need to be included in the regression model, the linearity of the relationships between the response and predictors, a test for possible omitted variables, and NGA fixed effects. As a result, we run many regressions, identify the “best model” (with the smallest Akaike Information Index, AIC) and sort the rest by increasing ∆AIC (difference from the best model). Once this model selection and testing phase is accomplished, the second phase uses nonparametric bootstrap to approximate the P-values.

All analyses were performed using R version 4.0.2. R-scripts and data files are published as a supplement to the article.

# Testing Bias in Aggregation of MilTech Variables

We further tested whether our results were impacted by the specific set of variables defined as part of the overall MilTech score or our aggregation procedure. We tested this by implementing a bootstrapping analysis. Instead of calculating MilTech as the sum of all six Warfare Components as we do for the primary analyses, we sampled from each of the WCs with replacement and then ran an exhaustive search regression with the response variable defined as a sum (thus, the WC ‘Project’, for example, could be counted twice and ‘Weapon’ be left out). Repeating the procedure 100 times we obtained 100 models sorted by AIC. The table below shows the proportion of these best models in which each factors failed to display a significant effect on the response variable:

**Table S1. Proportion of best models with significant effects on bootstrapped MilTech measures**

| **Variable** | **Proportion Significant** |
| --- | --- |
| MilTech | 0 |
| MilTech.sq | 0 |
| Scale | 96 |
| SocSoph | 98 |
| IronCav | 1 |
| Agri | 36 |
| WorldPop | 0 |
| Centrality | 0 |
| Phylogeny | 1 |

Thus we observe that the strongly supported terms from the primary analysis (those with large *t*-values) are either always selected, or omitted in just 1 case out of 100; namely, the autoregressive terms Miltech and MiTech.sq, the combined term IronCav, WorldPop, Centrality, and Phylogeny. Likewise, the two terms that were not selected in the best model using the non-bootstrapped data, Scale and SocSoph, were selected only in 2 or 4 cases, corresponding to the lack of effect these factors display in the primary analyses. The term that showed the most variability was Agri, which was part of the best model in only 64 cases (and left out in 36). Notably, as discussed in the main text *Dynamic Regressions Results*, while Agri appears as a significant predictor of subsequent levels of MilTech in the best models, it displays a lower *t-*value than the other significant factors (see main text Teble 1).

This bootstrapping analysis suggests that randomly subsampling from our various MilTech variables (WCs) has a negligible effect on our overall results, with the exception of Agri. The implication is that different WCs can substitute for each other with little loss of information, something that is also supported by comparing results using MilTech as the response variable versus using CoreMil (mentioned in the main text and detailed below). This is in line with our interpretation that early gains in the productivity of agriculture are a necessary component allowing for subsequent developments in terms of technological evolution as well as developing social complexity, but that once an suitable resource base has been reached, the evolution of military technologies are primarily driven by other factors highlighted in this article.

# Supplementary Results

## Basic Statistics and Cross-correlations

The chart below (Fig S2) provides two kinds of information: frequency distributions of various warfare characteristics (WCs) and cross-correlations between them, the overall MilTech, and calendar time. Several WCs show bimodality: Armor, Animal, and Defense. But the overall MilTech is less bimodal with peaks around 5 and 25.


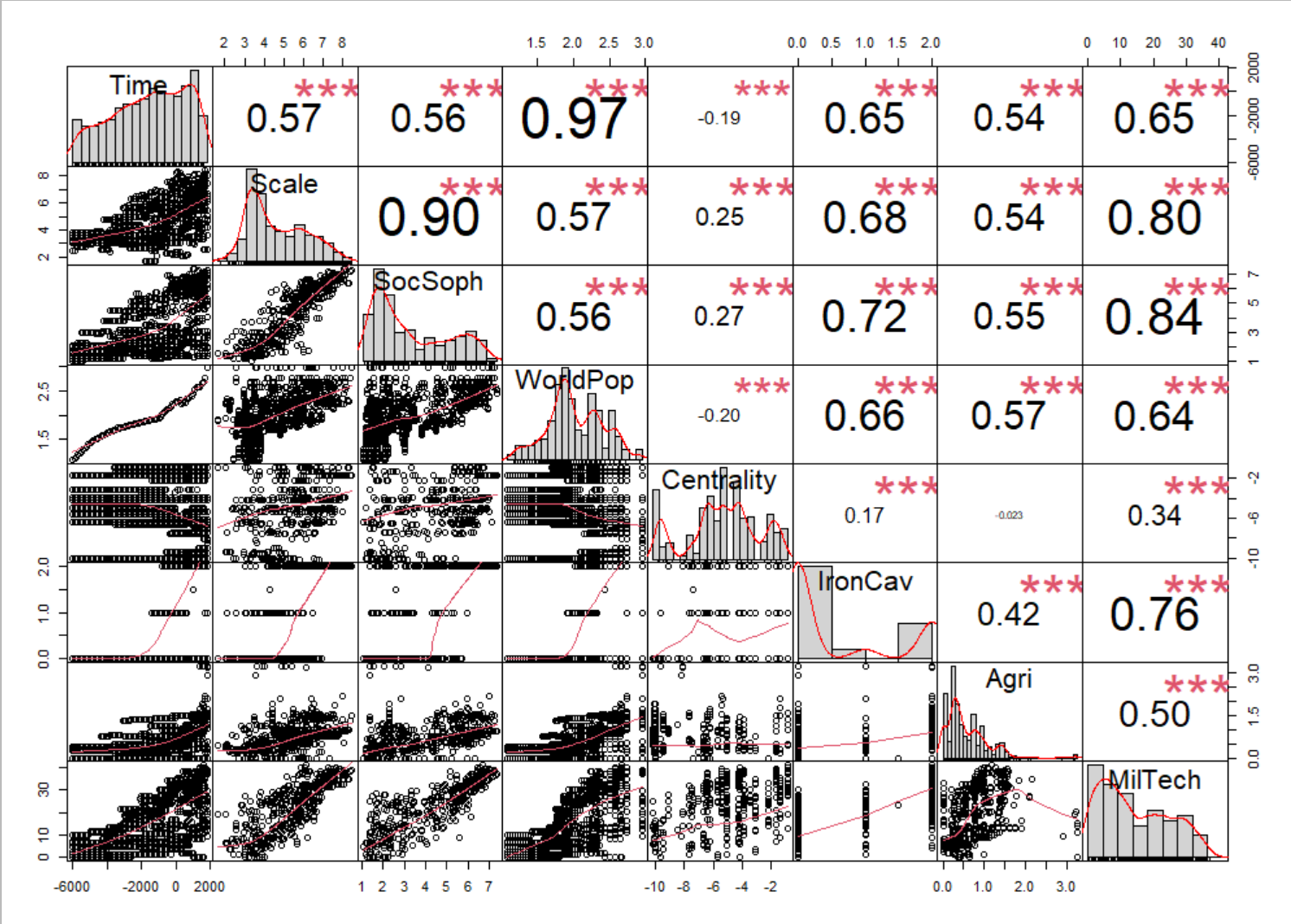


**Fig S2. Basic statistics for warfare characteristics.**

As expected, all WCs are closely correlated among each other and to the overall MilTech. Principal Component Analysis (see below) confirms these results. All WCs load on (are correlated with) the first principal component (PC) evenly, which explains 76 percent variation in the data. The second PC suggests two clusters of variables: Metal + Animal + Armor and Project + Weapon + Defense. Here is the breakdown of component loadings for this principal component analysis:

|  | PC1 | PC2 | PC3 | PC4 | PC5 | PC6 |
| --- | --- | --- | --- | --- | --- | --- |
| Standard deviation | 2.1158 | 0.66486 | 0.61489 | 0.58127 | 0.46146 | 0.3904 |
| Proportion of Variance | 0.7461 | 0.07367 | 0.06302 | 0.05631 | 0.03549 | 0.0254 |
| Cumulative Proportion | 0.7461 | 0.81978 | 0.88280 | 0.93911 | 0.97460 | 1.0000 |
| Standard deviations | (1, .., p=6) | |  |  |  |  |
| [1] 2.12 0.66 0.61 0.58 0.46 0.39 | | | | | | |

Here are the rotation (n x k) = (6 x 6) values:

|  | PC1 | PC2 | PC3 | PC4 | PC5 | PC6 |
| --- | --- | --- | --- | --- | --- | --- |
| Metal | 0.42 | -0.421 | 0.150 - | 0.099 | 0.784 - | 0.031 |
| Project | 0.40 | 0.052 | -0.092 | 0.896 | -0.048 | 0.152 |
| Weapon | 0.38 | 0.582 | 0.701 | -0.122 | -0.044 | -0.084 |
| Animal | 0.43 | -0.340 | 0.048 | -0.300 | -0.430 | 0.652 |
| Armor | 0.43 | -0.295 | -0.120 | -0.086 | -0.405 | -0.735 |
| Defense | 0.39 | 0.528 | -0.679 | -0.275 | 0.175 | 0.061 |

Our measure of MilTech is closely correlated with PC1 (regression *R*^2^ = 0.99), suggesting that it is appropriate to add equally weighted WCs together for an overall measure:

|  | Estimate | Sdt. Error | t value | Pr(?\|t) |
| --- | --- | --- | --- | --- |
| (Intercept) | 15.51692 | 0.02723 | 569.9 | <2e-16 *** |
| WPC1 | 5.38365 | 0.01288 | 418.1 | <2e-16 *** |

Signif. codes: 0 ‘***’ 0.001 ‘**’ 0.01 ‘*’ 0.05 ‘.’ 0.1 ‘ ’ 1

Residual standard error: 0.9302 on 1165 degrees of freedom

Multiple R-squared: 0.9934, Adjusted R-squared: 0.9934

F-statistic: 1.748e+05 on 1 and 1165 DF, p-value: < 2.2e-16


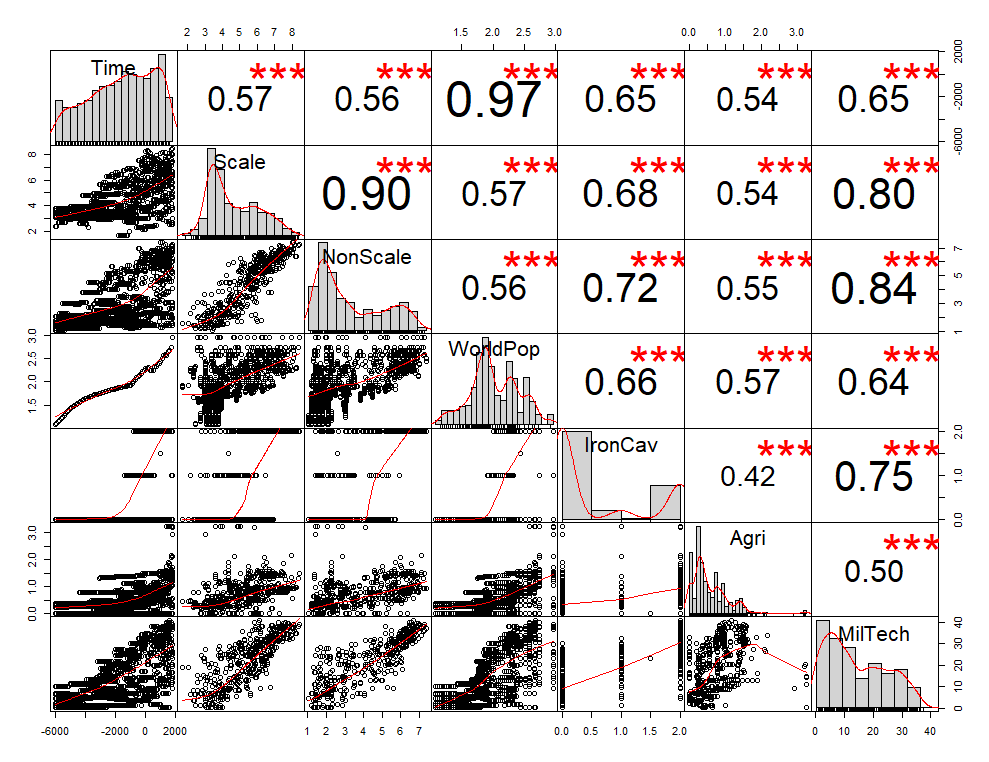


**Fig S3. Cross-correlations between MilTech and potential predictor variables.**

Next we examine the frequency distributions of potential predictors, and their cross-correlations with each other and with MilTech (Fig S3). Notable features include the observation that both the scale-related (Scale) and non-scale (SocSoph) aspects of social complexity have a bimodal distribution (just as MilTech). Furthermore, the correlations between these two variables and MilTech are high. The direction of causality that explains this correlations, of course, remains to be determined (see Dynamic Regression results below). Our measure of agricultural productivity (Agri) appears to be nonlinearly related to MilTech. This is another consideration we checked with our regression analysis.

Finally, we plot the time-line of military innovations (Fig S4). Note that this timeline represents not when a particular innovation has appeared anywhere in the world, but only the earliest time when it shows up in the data for one of the polities in the Seshat sample. For comparison, we also plot the log-transformed World Population. Generally, the two curves tend to rise in parallel, but they diverge during the last millennium. This divergence is probably due to the limitations of our MilTech measure, which doesn’t adequately capture the dramatic increase in the killing power that occurred after 1500 CE with the advent and spread of mortar and gunpowder weaponry, underrepresented in the Seshat sample which was constructed to focus on periods prior to the Industrial Revolution.


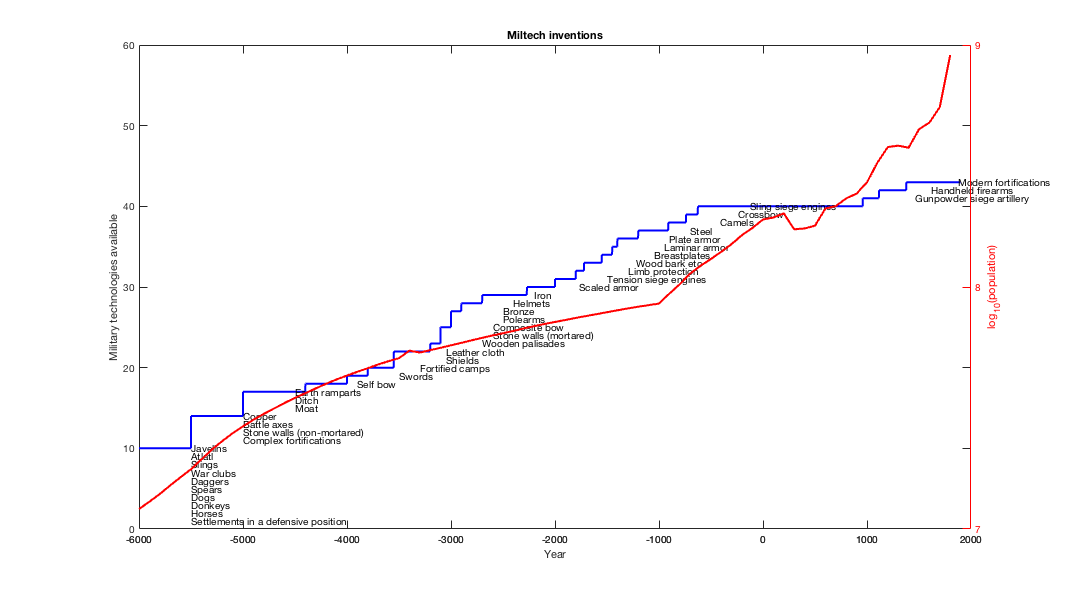


**Fig S4. The time line of MilTech innovations appearing in our dataset (blue stepped curve) plotted together with WorldPop (log-transformed world population, red curve).**

## Dynamic Regression Results: Preliminary Checks

We performed two preliminary checks to determine the set of variables used in exhaustive regressions. First, we investigated how the measure Centrality, based on the distance between each NGA and its nearest Silk Route node (explained above) compares with a direct estimate of the NGA effect. We then used the best supported predictor variable emerging from the exhaustive search (see below for this analysis ), included NGA as a categorical variable in the regression, and observed the following results:

|  | Estimate | Sdt. Error | t value | Pr(?\|t) |
| --- | --- | --- | --- | --- |
| (Intercept) | -0.053637 | 0.025404 | -2.111 | 0.034970 * |
| MilTech | 1.033588 | 0.031713 | 32.592 | < 2e-16 *** |
| MilTech.sq | -0.217127 | 0.030221 | -7.185 | 1.24e-12 *** |
| IronCav | 0.076807 | 0.015077 | 5.094 | 4.12e-07 *** |
| Agri | 0.008325 | 0.012274 | 0.678 | 0.497730 |
| WorldPop | 0.078316 | 0.014984 | 5.227 | 2.07e-07 *** |
| Phylogeny | 0.034999 | 0.009396 | 3.725 | 0.000205 *** |
| Big Island Hawaii | -0.046191 | 0.091554 | -0.505 | 0.613994 |
| Cahokia | -0.073721 | 0.052505 | -1.404 | 0.160579 |
| Cambodian Basin | -0.007986 | 0.048294 | -0.165 | 0.868689 |
| Central Java | -0.006831 | 0.053096 | -0.129 | 0.897662 |
| Crete | 0.087600 | 0.036169 | 2.422 | 0.015600 * |
| Cuzco | 0.035370 | 0.062642 | 0.565 | 0.572434 |
| Deccan | 0.050758 | 0.042407 | 1.197 | 0.231596 |
| Finger Lakes | 0.072871 | 0.098789 | 0.738 | 0.460889 |
| Galilee | 0.198918 | 0.067283 | 2.956 | 0.003179 ** |
| Ghanaian Coast | -0.104516 | 0.164296 | -0.636 | 0.524813 |
| Iceland | -0.077492 | 0.126265 | -0.614 | 0.539524 |
| Kachi Plain | 0.062651 | 0.036419 | 1.720 | 0.085668 . |
| Kansai | 0.057412 | 0.034577 | 1.660 | 0.097116 . |
| Kapuasi Basin | -0.248355 | 0.211394 | -1.175 | 0.240313 |
| Konya Plain | 0.134053 | 0.039142 | 3.425 | 0.000638 *** |
| Latium | 0.103925 | 0.040556 | 2.563 | 0.010525 * |
| Lena River Valley | -0.146003 | 0.112148 | -1.302 | 0.193233 |
| Lowland Andes | -0.283217 | 0.151670 | -1.867 | 0.062124 . |
| Middle Ganga | 0.039011 | 0.036632 | 1.065 | 0.287130 |
| Middle Yellow River Valley | 0.161282 | 0.038194 | 4.223 | 2.61e-05 *** |
| Niger Inland Delta | -0.116259 | 0.059390 | -1.958 | 0.050536 . |
| North Colombia | -0.082440 | 0.069044 | -1.194 | 0.232731 |
| Orkhon Valley | -0.040901 | 0.052743 | -0.775 | 0.438225 |
| Paris Basin | 0.099040 | 0.044683 | 2.217 | 0.026862 * |
| Sogdiana | 0.047158 | 0.041226 | 1.144 | 0.252920 |
| Southern Mesopotamia | 0.103160 | 0.039112 | 2.638 | 0.008469 ** |
| Susiana | 0.075411 | 0.037507 | 2.011 | 0.044614 * |
| Upper Egypt | 0.110168 | 0.045903 | 2.400 | 0.016560 * |
| Valley of Oaxaca | -0.076598 | 0.047434 | -1.615 | 0.106633 |
| Yemeni Coastal Plain | -0.038012 | 0.039497 | -0.962 | 0.336064 |

Signif. codes: 0 ‘***’ 0.001 ‘**’ 0.01 ‘*’ 0.05 ‘.’ 0.1 ‘ ’ 1

(Dispersion parameter for gaussian family taken to be 0.0430152)

Null deviance: 1131.000 on 1131 degrees of freedom

Residual deviance: 47.102 on 1095 degrees of freedom

AIC: -310.64

Plotting the t-values associated with each NGA against the NGA’s Centrality suggests that this measure provides an imperfect, but reasonably accurate measure of how NGA location affects the evolution of MilTech (Figs S5 and S6; note that Centrality captures around half of the variance in the spatial location effect).

**
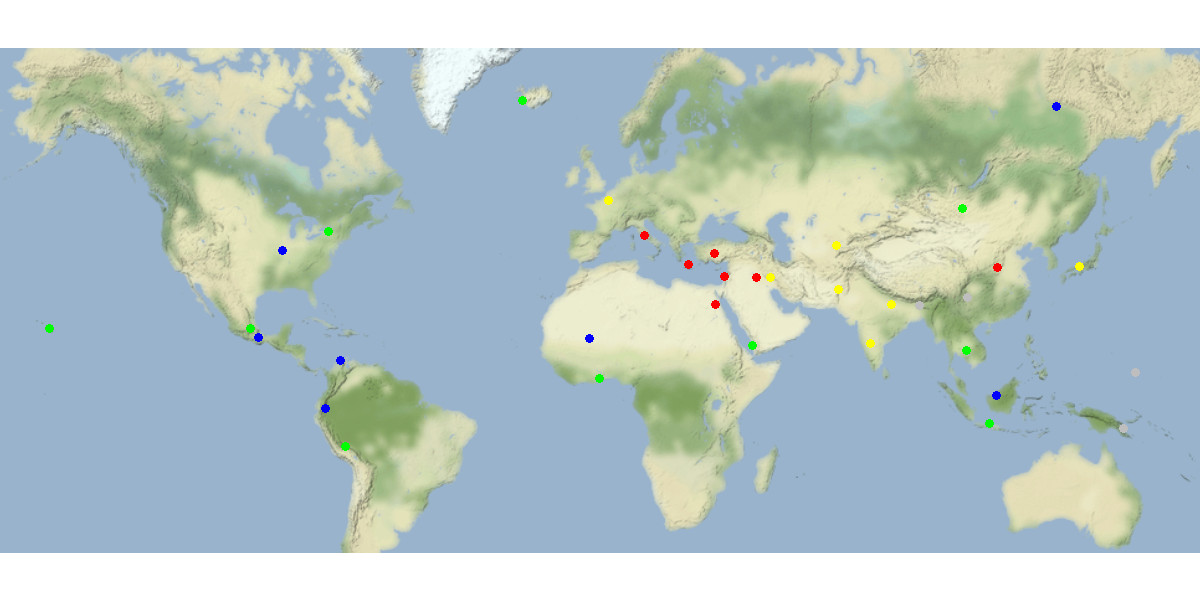
**

**Fig S5. NGA locations plotted on the world map, color-coded for the location (NGA) fixed effect on the development of MilTech in the NGA. Red = strongly positive, yellow = mildly positive, green = mildly negative, blue = strongly negative, and grey = not enough data to estimate the NGA effect.**


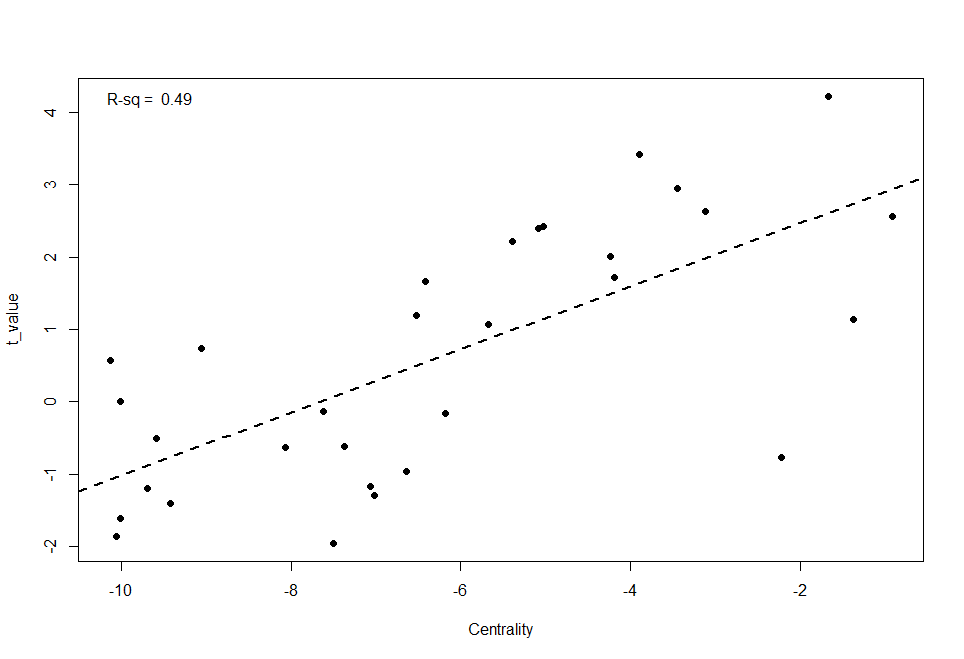


**Fig S6. Relationship between Centrality (measured as –*d^θ^* , where *d* is the distance to the nearest Silk Route node and *θ* = 0.25) and the NGA fixed effect (quantified by the t_value).**

When we replace the NGA fixed effect with Centrality, we obtain a much better fitting model:

|  | Estimate | Sdt. Error | t value | Pr(?\|t) |
| --- | --- | --- | --- | --- |
| (Intercept) | -2.892e-16 | 6.200e-03 | 0.000 | 1.000000 |
| MilTech | 1.043e+00 | 2.477e-02 | 42.114 | < 2e-16 *** |
| MilTech.sq | -1.754e-01 | 2.556e-02 | -6.862 | 1.12e-11 *** |
| IronCav | 4.678e-02 | 1.177e-02 | 3.973 | 7.55e-05 *** |
| Agri | 1.985e-02 | 7.810e-03 | 2.542 | 0.011144 * |
| WorldPop | 3.893e-02 | 1.111e-02 | 3.505 | 0.000475 *** |
| Centrality | 2.734e-02 | 8.102e-03 | 3.375 | 0.000763 *** |
| Phylogeny | 3.726e-02 | 8.305e-03 | 4.486 | 8.01e-06 *** |

Signif. codes: 0 ‘***’ 0.001 ‘**’ 0.01 ‘*’ 0.05 ‘.’ 0.1 ‘ ’ 1

(Dispersion parameter for gaussian family taken to be 0.04351352)

Null deviance: 1131.000 on 1131 degrees of freedom

Residual deviance: 48.909 on 1124 degrees of freedom

AIC: -326.01

Because the AIC the model with NGA fixed effects is much larger than the AIC for the model with Centrality (lower AIC indicates a better model), it made sense to proceed with the simpler and better supported model; thus, in the main text we report only results using Centrality as our measure of NGA location-related effects.

The second check was on how the synthetic variable, IronCav, preformed against using Iron and Cavalry separately. Running regressions with all combinations of terms, sorting them by AIC, and retaining only those models for which ∆AIC < 2, we obtained the following results:

| **MilTech** | **MilTech.sq** | **Scale** | **SocSoph** | **Iron** | **Cavalry** | **IronCav** | **Agri** | **WorldPop** | **Centr.** | **Phylog.** | **∆AIC** |
| --- | --- | --- | --- | --- | --- | --- | --- | --- | --- | --- | --- |
| 42.11 | -6.86 |  |  |  |  | 3.97 | 2.54 | 3.50 | 3.38 | 4.49 | 0.00 |
| 42.27 | -6.64 |  |  | 3.87 |  |  | 2.43 | 3.77 | 3.48 | 4.59 | 0.80 |
| 41.86 | -6.67 |  |  | 2.57 | 1.12 |  | 2.52 | 3.50 | 3.39 | 4.45 | 1.53 |
| 41.86 | -6.67 |  |  |  | -0.68 | 2.57 | 2.52 | 3.50 | 3.39 | 4.45 | 1.53 |
| 41.86 | -6.67 |  |  | 2.57 | 1.12 | 2.52 | 3.50 | 3.39 | 4.45 | 0.00 | 1.53 |
| 41.86 | -6.67 |  |  | 0.68 |  | 1.12 | 2.52 | 3.50 | 3.39 | 4.45 | 1.53 |
| 41.83 | -6.70 | -0.61 |  |  |  | 4.02 | 2.61 | 3.51 | 3.40 | 4.43 | 1.63 |
| 41.77 | -6.74 |  | 0.20 |  |  | 3.92 | 2.38 | 3.51 | 3.37 | 4.47 | 1.96 |
| 41.97 | -6.54 |  | 0.44 | 3.83 |  |  | 2.21 | 3.78 | 3.47 | 4.56 | 2.61 |
| 42.02 | -6.47 | -0.28 |  | 3.88 |  |  | 2.43 | 3.77 | 3.49 | 4.58 | 2.72 |
| 41.74 | -6.73 | -0.98 | 0.79 |  |  | 3.97 | 2.43 | 3.57 | 3.42 | 4.32 | 3.00 |

Each row in this table corresponds to a separate model specification. Empty cells indicate that the particular predictor was not part of the model. Models are sorted by AIC from the best (lowest AIC, ∆AIC = 0) to worse. Centr. = Centrality, Phylog. = Phylogeny

We observe that the model that includes the synthetic variable IronCav has a lower AIC than models including Iron or Cavalry (or both). In the following analyses we thus use only IronCav and focus on models using this synthetic variable in the main text.

## Dynamic Regression Results: Exhaustive Search

After these initial checks, we ran regressions for all combinations of predictors (but without Iron and Cavalry, using instead IronCav and using Centrality rather than the NGA fixed effects terms). The results are (also reported in Table 2 of the main article):

| **MilTech** | **MilTech.sq** | **Scale** | **SocSoph** | **IronCav** | **Agri** | **WorldPop** | **Centrality** | **Phylogeny** | **∆AIC** |
| --- | --- | --- | --- | --- | --- | --- | --- | --- | --- |
| 42.11 | -6.86 |  |  | 3.97 | 2.54 | 3.50 | 3.38 | 4.49 | 0.00 |
| 41.83 | -6.70 | -0.61 |  | 4.02 | 2.61 | 3.51 | 3.40 | 4.43 | 1.63 |
| 41.77 | -6.74 |  | 0.20 | 3.92 | 2.38 | 3.51 | 3.37 | 4.47 | 1.96 |
| 41.74 | -6.73 | -0.98 | 0.79 | 3.97 | 2.43 | 3.57 | 3.42 | 4.32 | 3.00 |

These results indicate that neither Scale nor the non-scale aspects of complexity (SocSoph) have significant effects on the response variable.

We then checked that the main result of our analysis is not changed when we use CoreMil instead of MilTech. This is a measure of MilTech aggregated in the same way as described above for the primary analysis, but omitting the Metals and Military Animals WCs, since there are possible confounds between these measures and the Iron and Cavalry variables, respectively, as described in the main text. This analysis reveals that the main results indeed are still found:

| **CoreMil** | **CoreMil.sq** | **Scale** | **SocSoph** | **IronCav** | **Agri** | **WorldPop** | **Centrality** | **Phylogeny** | **∆AIC** |
| --- | --- | --- | --- | --- | --- | --- | --- | --- | --- |
| 39.67 | -6.63 |  |  | 3.80 | 3.16 | 3.47 | 3.42 | 4.47 | 0.00 |
| 39.37 | -6.58 |  | 0.49 | 3.64 | 2.91 | 3.49 | 3.40 | 4.43 | 1.76 |
| 39.30 | -6.52 | -0.25 |  | 3.78 | 3.13 | 3.47 | 3.42 | 4.44 | 1.94 |

Returning to MilTech as the response variable, the best model identified by AIC is as follows (also reported in Table 1 of the main article):

|  | Estimate | Sdt. Error | t value | Pr(?\|t) |
| --- | --- | --- | --- | --- |
| (Intercept) | -2.892e-16 | 6.200e-03 | 0.000 | 1.000000 |
| MilTech | 1.043e+00 | 2.477e-02 | 42.114 | < 2e-16 *** |
| MilTech.sq | -1.754e-01 | 2.556e-02 | -6.862 | 1.12e-11 *** |
| IronCav | 4.678e-02 | 1.177e-02 | 3.973 |  |
| Agri | 1.985e-02 | 7.810e-03 | 2.542 | 0.011144 * |
| WorldPop | 3.893e-02 | 1.111e-02 | 3.505 | 0.000475 *** |
| Centrality | 2.734e-02 | 8.102e-03 | 3.375 | 0.000763 *** |
| Phylogeny | 3.726e-02 | 8.305e-03 | 4.486 | 8.01e-06 *** |

Signif. codes: 0 ‘***’ 0.001 ‘**’ 0.01 ‘*’ 0.05 ‘.’ 0.1 ‘ ’ 1

Residual standard error: 0.2086 on 1124 degrees of freedom

Multiple R-squared: 0.9568, Adjusted R-squared: 0.9565

F-statistic: 3553 on 7 and 1124 DF, p-value: < 2.2e-16

### Checking for autocorrelations and nonlinearities

A check for Space indicates that the addition of this term does not better the regression results:

|  | Estimate | Std. Error | t value | Pr(>\|t\|) |
| --- | --- | --- | --- | --- |
| (Intercept) | -2.882e-16 | 6.201e-03 | 0.000 | 1.000000 |
| MilTech | 1.038e+00 | 2.573e-02 | 40.335 | < 2e-16 *** |
| MilTech.sq | -1.727e-01 | 2.581e-02 | -6.690 | 3.51e-11 *** |
| IronCav | 4.911e-02 | 1.217e-02 | 4.035 | 5.82e-05 *** |
| Agri | 1.907e-02 | 7.880e-03 | 2.419 | 0.015702 * |
| WorldPop | 3.938e-02 | 1.113e-02 | 3.540 | 0.000417 *** |
| Centrality | 2.596e-02 | 8.305e-03 | 3.126 | 0.001818 ** |
| Phylogeny | 3.564e-02 | 8.575e-03 | 4.156 | 3.48e-05 *** |
| Space | 5.935e-03 | 7.809e-03 | 0.760 | 0.447437 |

Signif. codes: 0 ‘***’ 0.001 ‘**’ 0.01 ‘*’ 0.05 ‘.’ 0.1 ‘ ’ 1

Residual standard error: 0.2086 on 1123 degrees of freedom

Multiple R-squared: 0.9568, Adjusted R-squared: 0.9565

F-statistic: 3107 on 8 and 1123 DF, p-value: < 2.2e-16

A check for Lag2 (*Y_i,t_* _– 2_) similarly does not find a significant effect. Furthermore, adding Lag2 does not change the regression results substantially, with the exception of the Agri estimate that becomes weaker.

|  | Estimate | Std. Error | t value | Pr(>\|t\|) |
| --- | --- | --- | --- | --- |
| (Intercept) | -0.002595 | 0.006237 | -0.416 | 0.677472 |
| MilTech | 0.990725 | 0.036869 | 26.872 | < 2e-16 *** |
| MilTech.sq | -0.146495 | 0.025661 | -5.709 | 1.47e-08 *** |
| IronCav | 0.042096 | 0.011837 | 3.556 | 0.000393 *** |
| Agri | 0.017340 | 0.007989 | 2.171 | 0.030188 * |
| WorldPop | 0.041428 | 0.011426 | 3.626 | 0.000302 *** |
| Centrality | 0.023659 | 0.008141 | 2.906 | 0.003735 ** |
| Phylogeny | 0.016529 | 0.009194 | 1.798 | 0.072497 . |
| Lag2 | 0.040431 | 0.028773 | 1.405 | 0.160269 |

Signif. codes: 0 ‘***’ 0.001 ‘**’ 0.01 ‘*’ 0.05 ‘.’ 0.1 ‘ ’ 1

Residual standard error: 0.203 on 1062 degrees of freedom

(61 observations deleted due to missingness)

Multiple R-squared: 0.9582, Adjusted R-squared: 0.9579

F-statistic: 3047 on 8 and 1062 DF, p-value: < 2.2e-16

The plot of MilTech against Agri suggested that the relationship between these two variables may be nonlinear (see *Basic Statistics and Cross-Correlations* above). We check for such a possible nonlinearity effect by adding a quadratic term for Agri. However, this results in lack of support for the quadratic Agri term

|  | Estimate | Std. Error | t value | Pr(>\|t\|) |
| --- | --- | --- | --- | --- |
| (Intercept) | 0.001338 | 0.006915 | 0.194 | 0.846595 |
| MilTech | 1.042318 | 0.024867 | 41.916 | < 2e-16 *** |
| MilTech.sq | -0.175859 | 0.025589 | -6.872 | 1.04e-11 *** |
| IronCav | 0.047232 | 0.011824 | 3.995 | 6.90e-05 *** |
| Agri | 0.022611 | 0.010035 | 2.253 | 0.024446 * |
| Agri.sq | -0.001339 | 0.003061 | -0.438 | 0.661816 |
| WorldPop | 0.038996 | 0.011113 | 3.509 | 0.000467 *** |
| Centrality | 0.027310 | 0.008105 | 3.370 | 0.000778 *** |
| Phylogeny | 0.036962 | 0.008335 | 4.434 | 1.01e-05 *** |

Signif. codes: 0 ‘***’ 0.001 ‘**’ 0.01 ‘*’ 0.05 ‘.’ 0.1 ‘ ’ 1

Residual standard error: 0.2087 on 1123 degrees of freedom

Multiple R-squared: 0.9568, Adjusted R-squared: 0.9565

F-statistic: 3106 on 8 and 1123 DF, p-value: < 2.2e-16

### Diagnostics of Dynamic Regression Results

Standard diagnostic plots look good, with the exception of the Normal Q-Q plot, which suggests substantial deviations from normality:


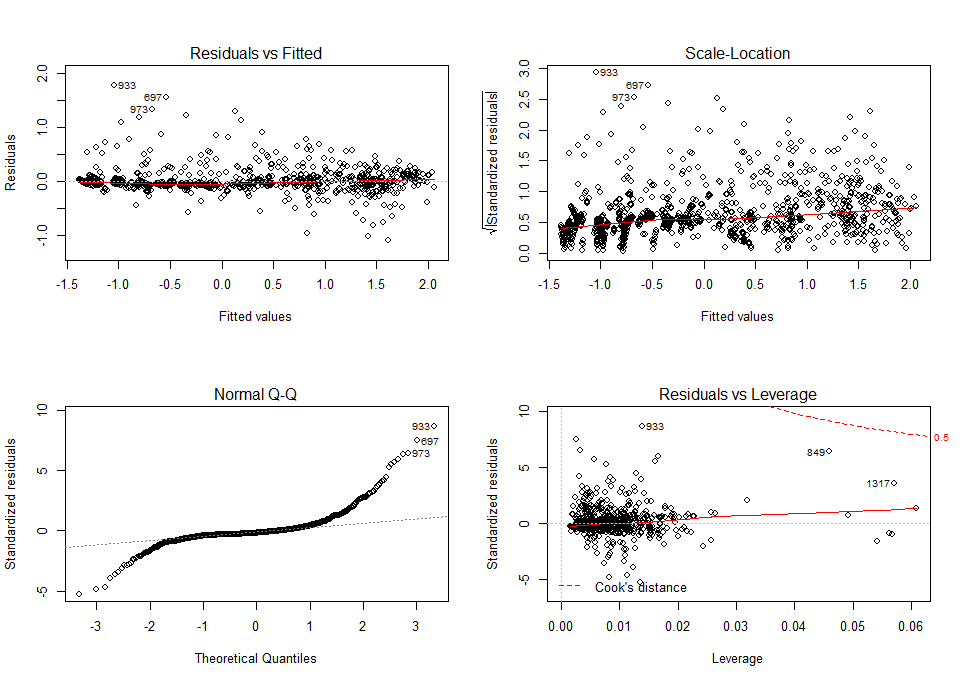


**Fig S7. Diagnostic plots for the “best” regression model.**

Plotting the frequency distribution of residuals (Fig S8) together with the predicted Normal distribution (the curve) suggests that the residual distribution is more leptokurtic (with long tails) than a Gaussian:


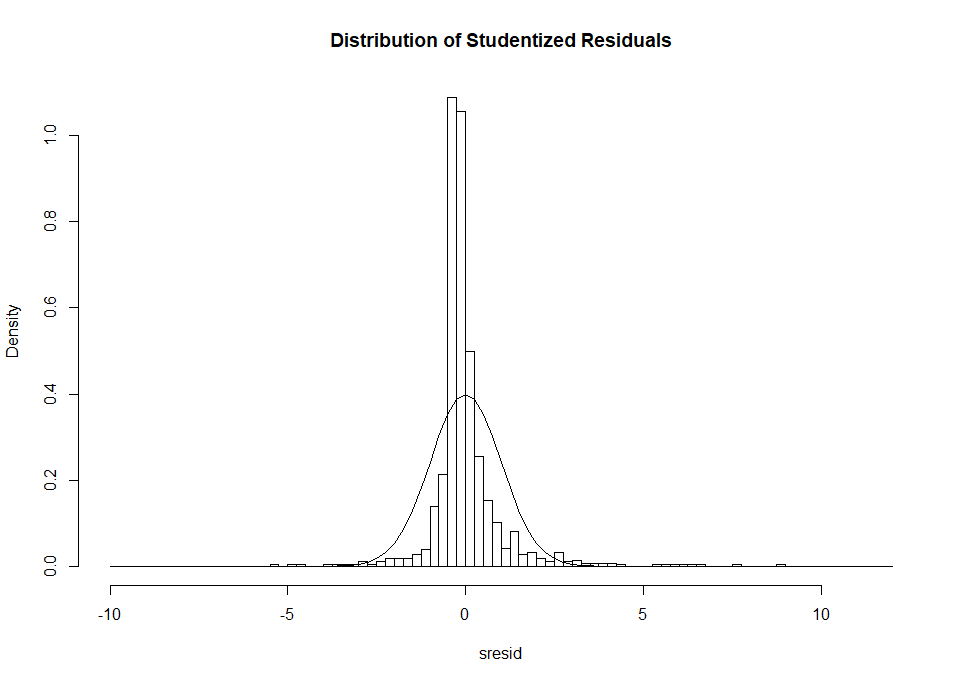


**Fig S8. Frequency distribution of studentized residuals (the bar chart) against the theoretical Gaussian distribution (the curve).**

This observation suggests that the *P*-values calculated by assuming normality would be overly liberal (a tendency to suggest a greater degree of statistical significance than warranted by the data).

### Results of Nonparametric Bootstrap

Nonparametric bootstrap, which does not make this assumption, approximates the distribution of regression coefficients as follows:


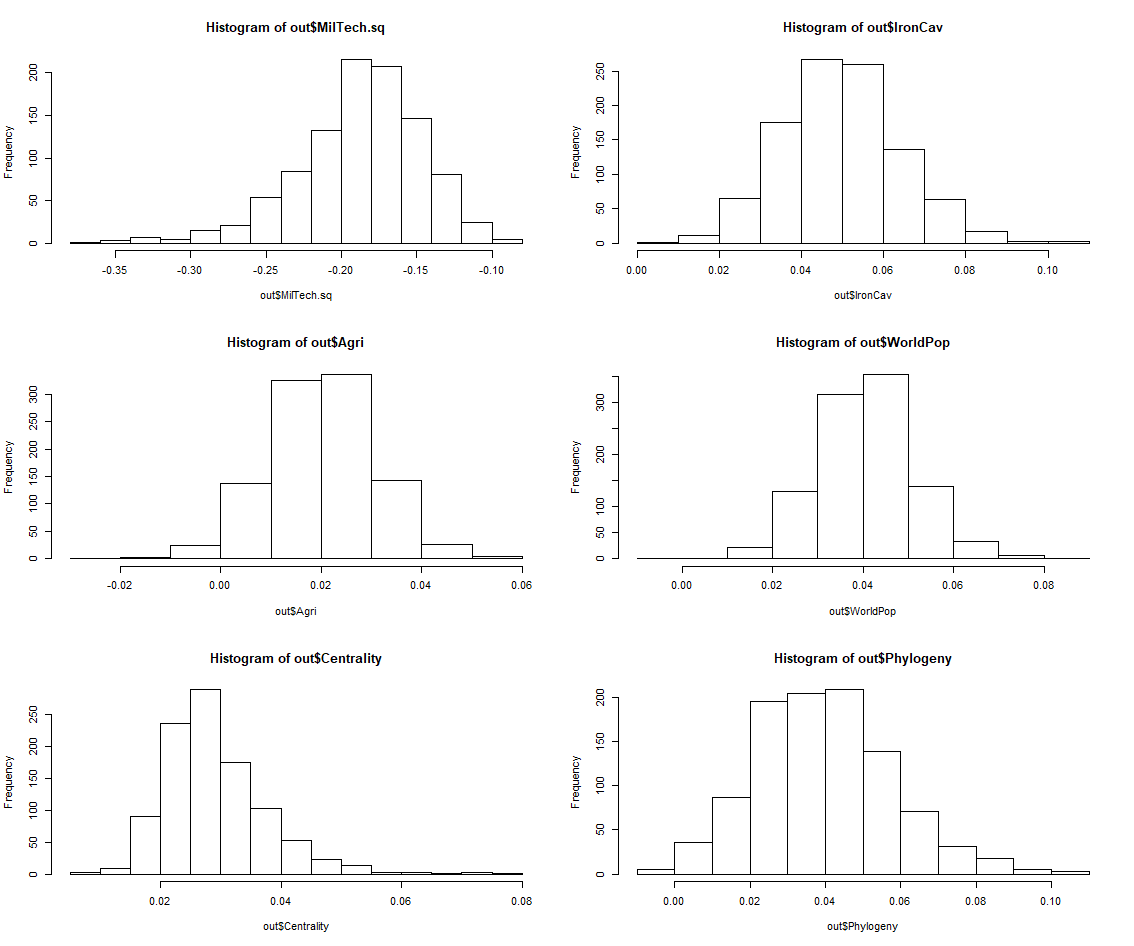


**Fig S9. Frequency distributions of 1000 bootstrap values of regression coefficients associated with the predictor variables.**

The bootstrapped *P*-values are reported in Table 1 of the main article.

# Investigating the Effect of Alternative MilTech Coding

The final robustness test that we perform is checking how our results are affected by the use of the “strong evidence” rule. To do this, we implemented an alternative coding that does not convert unknowns into inferred absences. The main result of this rule is that a substantial proportion of the dataset ends up omitted from the analysis (reducing the sample size from *n* = 1132 to *n* = 606). The results of exhaustive searching indicate that omitting most of the dataset results in dropping Agri and weakening the statistical significance of the terms in the best supported model, though these terms are still positive predictors of subsequent MilTech in the best model:

| **MilTech** | **MilTech.sq** | **Scale** | **SocSoph** | **IronCav** | **Agri** | **WorldPop** | **Centr.** | **Phylogeny** | **∆AIC** |
| --- | --- | --- | --- | --- | --- | --- | --- | --- | --- |
| 28.66 | -4.94 |  |  | 1.78 |  | 4.55 | 4.06 | 4.68 |  |
| 28.44 | -5.05 |  | 1.01 | 1.51 |  | 4.19 | 3.57 | 4.72 | 0.96 |
| 28.96 | -4.61 |  |  |  |  | 5.29 | 4.02 | 5.31 | 1.19 |
| 28.89 | -4.82 |  | 1.38 |  |  | 4.64 | 3.45 | 5.30 | 1.27 |
| 28.56 | -4.95 |  |  | 1.79 | 0.41 | 4.30 | 4.03 | 4.63 | 1.83 |
| 28.54 | -4.93 | 0.28 |  | 1.69 |  | 4.36 | 3.73 | 4.67 | 1.92 |

However, most of the missing values are concentrated in the Armor and Defenses variables. If we define a reduced measure of MilTech that only sums the first four WCs (Metal, Projectile, Weapon, and Animal; refer to it as MilTech_alt), then the sample size is much less affected (*n* = 1120) and results are nearly identical to the full MilTech_main measure (the only partial exception is that statistical support for Phylogeny weakens):

| **MilTech** | **MilTech.sq** | **Scale** | **SocSoph** | **IronCav** | **Agri** | **WorldPop** | **Centr** | **Phylogeny** | **∆AIC** |
| --- | --- | --- | --- | --- | --- | --- | --- | --- | --- |
| 41.97 | -6.02 | -1.74 |  | 3.61 | 2.11 | 6.02 | 4.60 | 1.93 | 0.00 |
| 41.90 | -6.24 |  |  | 3.36 | 1.73 | 5.80 | 4.29 | 2.00 | 1.04 |
| 41.94 | -5.88 |  | -1.34 | 3.53 | 2.05 | 5.93 | 4.49 | 2.10 | 1.22 |
| 42.00 | -5.94 | -1.81 |  | 4.59 | 2.40 | 5.88 | 4.69 |  | 1.75 |
| 41.95 | -5.90 | -1.13 | -0.28 | 3.62 | 2.13 | 6.02 | 4.59 | 1.95 | 1.92 |

A plot of MilTech_alt against MilTech_main (Fig S10) indicates that these two measures are closely correlated (*R*^2^ = 0.95), which explains why the regression results are so similar.
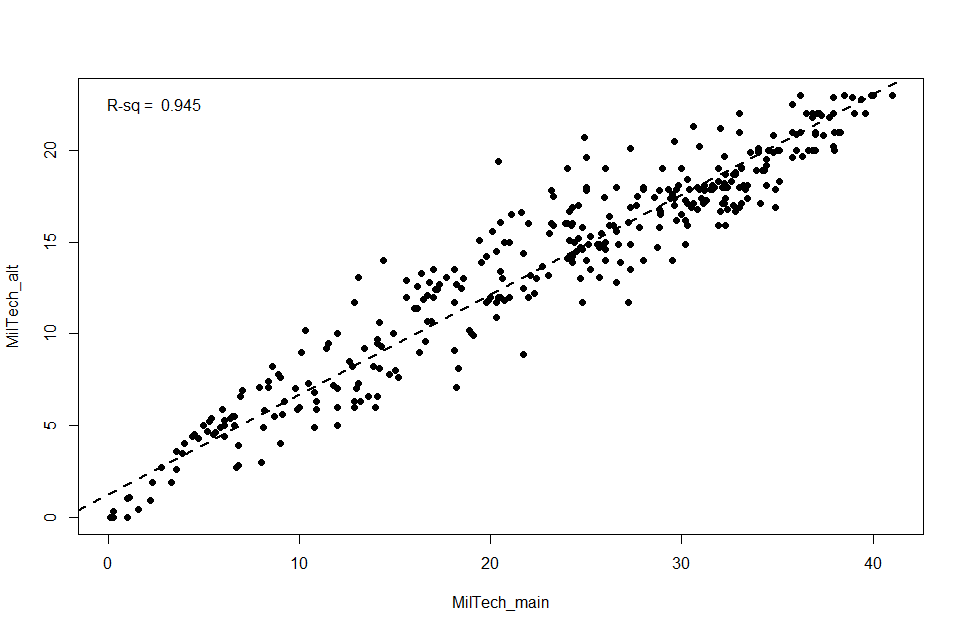


**Fig S10. Comparison of two MilTech measures. MilTech_main is a measure using the strong evidence rule and aggregating all six WCs, while MilTech_alt is a measure not using strong evidence and aggregating the four more fully coded WCs.**

As we saw above (Fig S2), all six WCs are strongly correlated with each other. This means that each of them can serve as a decent proxy for others, while four of them together result in an excellent proxy for a measure of MilTech utilizing all six.

The overall conclusion from these checks is that our main results are robust with respect to using, or not, the “strong evidence” rule.

**References**

1. Turchin P, Brennan R, Currie T, Feeney K, Francois P, Hoyer D, et al. Seshat: The Global History Databank. Cliodynamics. 2015;6:77-107.

2. Turchin P, Currie TE, Whitehouse H, Francois P, Feeney K, Mullins D, et al. Quantitative historical analysis uncovers a single dimension of complexity that structures global variation in human social organization. Proc Natl Acad Sci U S A. 2018;115(2):e144-e51.

3. Murdock GP. Ethnographic Atlas. Pittsburgh University of Pittsburgh Press; 1967.

4. Murdock GP, White DR. Standard Cross-Cultural Sample. Ethnology. 1969;8(4):329–69.

5. Peregrine PN. Atlas of Cultural Evolution. World Cultures. 2003;14(1):2-88.

6. Turchin P, Hoyer D, Bennett J, Basava K, Cioni E, Francois P, et al. The Equinox2020 Seshat Data Release. Cliodynamics. 2020;11(1):41-50.

7. Turchin P. Fitting Dynamic Regression Models to Seshat Data. Cliodynamics. 2018;9:25-58.

8. Chase-Dunn CK, Hall TD. Rise and demise: comparing world-systems. Boulder, CO: Westview Press; 1997. xi, 322 p.

9. Korotayev A, Malkov A, Khaltourina D. Introduction to Social Macrodynamics: Secular Cycles and Millennial Trends. Moscow: URSS; 2006.

10. Korotayev A. Compact mathematical models of world system development, and how they can help us to clarify our understanding of globalization processes. In: Modelski G, Devezas T, Thompson WR, editors. Globalization as Evolutionary Process: Modeling Global Change. London: Routledge; 2008. p. 133-60.

11. Williams T. The Silk Roads: An Icomos Thematic Study. International Council of Monuments and Sites. 2014.

12. Beckwith CI. Empires of the Silk Road: A History of Cenrtal Eurasia from the Bronze Age to the Present. Princeton: Princeton University Press; 2009.

13. Christian D. Silk Roads or Steppe Roads? The Silk Roads in World History. Journal of World History. 2000;11(1):1-26.

14. McLaughlin R. The Roman Empire and the Silk Routes: The Ancient World Economy & the Empires of Parthia, Central Asia & Han China: Pen and Sword; 2016.

15. Rubin DB. Multiple Imputation for Nonresponse in Surveys. New York: Wiley; 1987.

16. Eff EA, Dow MM. How to deal with missing data and Galton’s problem in cross-cultural survey research: a primer for R. Structure and Dynamics. 2009;3(3):Article 1.

17. Yuan YC. Multiple Imputation for Missing Data: Concepts and New Development (Version 9.0). Rockville, MD: SAS Institute; 2011.
