# Supplementary material for: Rise of the war machines: Charting the evolution of military technologies from the Neolithic to the Industrial Revolution
Source: PLoS One. 2021 Oct 20;16(10):e0258161. doi: 10.1371/journal.pone.0258161 (PMC8528290; doi:10.1371/journal.pone.0258161)
Supplement: Attachment — Submitted filename: PONE-D-21-17737 comments.docx [file pone.0258161.s003.docx]

This is a valuable paper, making a real contribution to a significant debate. My one reservation is that I think this kind of large-n survey usually has to be combined with more detailed work to explain something as complicated as military technologies. Military historians typically find that technology is only one part of effectiveness, and not always the most important part. The most interesting result in this paper is the lack of fit between SocSoph and technological gains (lines 455-56, 532-36), but I wonder how much of this is because technologies are little use without appropriate doctrine (the central point in Stephen Biddle’s book *Military Power* [2004]). Fig. 5 illustrates this—looking at the scores for Latium around 2kya, they’re not very different from other societies, because Roman military technology wasn’t actually that different from other Mediterranean, Middle Eastern, and European societies (as measured in the categories at lines 287-98 in the supporting material); however, the Roman army’s ability to apply these technologies transformed what they meant in practical terms, and that was probably determined primarily by Roman SocSoph. I also immediately think of the classical Greeks, whose military technologies were very ordinary but their application of them was extraordinary.

One difficulty for large-n surveys is that while technological categories are relatively easy to identify in the archaeological record, military doctrines, logistics, tactical skill, organizational structures, and ability to learn aren’t; and even when ancient and medieval writers describe such doctrines, there’s no obvious way to score them. This paper should absolutely be published, because there’ve been few (if any) large-n surveys of this level of sophistication, but the main point it illustrates is perhaps that large-n surveys are only the beginning of the study of warfare, and we have to follow up on their results with analytic narratives and case studies.

I also thought that the paper should distinguish more between technological innovation and diffusion. I’m currently reading two case studies of the Comanches, who probably had the best light cavalry in the world in the 18th century. They didn’t invent light cavalry, because there were no horses in North America to invent it with before the Spaniards brought them, but they did adopt and then adapt horse-herding and perfect styles of mounted fighting much better than the Apaches or even the Spaniards and Mexicans. They didn’t adopt or adapt firearms because their light-cavalry tactics made muzzle-loading muskets irrelevant. The Comanches were eventually defeated because Texans adopted and adapted Comanche tactics in the 1830s and combined them with revolvers and, later, breech-loading rifles that had been invented on the US East Coast. Neither the Comanche nor the Texans could have invented revolvers and rifles themselves, but the US Army failed to figure out how to use the new technologies until Texans put them together with tactics in new ways; but the final Comanche defeat in the 1870s depended on the US Army learning from the Texans and then exploiting the scale of US federal infrastructure.

These points aren’t just details that can be subsumed within a large-n model; we’re missing what really happened if we only see the coarse-grained technological/geographical narrative. The paper should be clearer that while a large-n survey is a necessary condition for a good explanation, it’s not sufficient, and should be treated as a starting point for other kinds of analysis.

One final detail: the throwaway comment on naval warfare on line 69 isn’t adequate.
